# *Babesia* in a Nonsplenectomized Patient Requiring Exchange Transfusion

**DOI:** 10.1155/2015/405263

**Published:** 2015-11-29

**Authors:** Dikshya Sharma, Bindu Mudduluru, Elias Moussaly, Neville Mobarakai, Matthew Hurford

**Affiliations:** Staten Island University Hospital, Staten Island, NY, USA

## Abstract

Babesiosis is a tick born zoonosis caused by red blood cell parasites of the genus* Babesia*. It is caused predominantly by* B. microti* and* B. divergens*,* microti* being more common in the US. The parasites are transmitted by* Ixodes* tick to their host but infection can also spread by blood transfusion and perinatally. Clinical manifestations vary from subclinical infection to fulminating disease depending upon the immune status of the patient. About half of patients, hospitalized with babesiosis, develop complication with fatality rates of 6 to 9% which increase up to 21% among those with immunosuppression. A case of 58-year-old previously healthy man, infected by* B*.* microti*, was reported on 2000 who presented with severe disease characterized by severe anemia, DIC, and renal and respiratory failure. First case of overwhelming septic shock without respiratory involvement due to babesiosis in a healthy patient with an intact spleen was published in a case report on 2011. Since our patient here is an immunocompetent healthy male with intact spleen presenting with severe babesiosis requiring exchange transfusion, this presentation of* Babesia* is rare and warrants further study.

## 1. Introduction

*Babesia* is among the most ubiquitous blood parasites and maintains their life cycle via exchange between* Ixodes* ticks and various mammals. Since its recognition on Nantucket Island and Cape Cod, Massachusetts, USA, during the 1970s, human babesiosis from* B. microti* infection has become a public health threat in several states.

## 2. Case

A 53-year-old male with no significant past medical history presented with 3-day history of fever, generalized weakness, cold sweats, nausea, and headache. He resides in Staten Island, New York, and is a wedding photographer by profession which requires him to travel around woods of upstate New York. He also admits working in his backyard for 2 weeks. He has two dogs and recalls no sick contact in the family, no insect or tick bite, or no recent travel outside United States. He is an occasional drinker and neither reports drug abuse nor has any history suggestive of functional or anatomical asplenia. In his primary doctor's office, he was found to be hypotensive and tachycardic with blood pressure of 90/60 and pulse of 101. Labs revealed platelet count of 41, total bilirubin of 3.2, and AST/ALT of 115 and 106 IU/L, respectively. He had a normal white and red blood cell count. A blood smear was positive for* B. microti*. His PMD requested him to report to emergency room.

In ER, physical examination showed a well-built healthy man in no acute distress. His vitals were stable. Physical examination was pertinent for jaundiced sclera and a reticular rash on his left arm consitent with livedo reticularis. A repeated blood work was sent that showed hemoglobin of 13.2 g/dL, platelet counts of 45 L, AST of 148 IU/L, ALT of 124 IU/L, and total billirubi of 3.4 mg/dL. High lactate dehydrogenase of 699 IU/L and decreased haptoglobulin of <3 were suggestive of intravascular hemolysis. He had negative hepatitis panel and HIV status.

**Figure 1:**
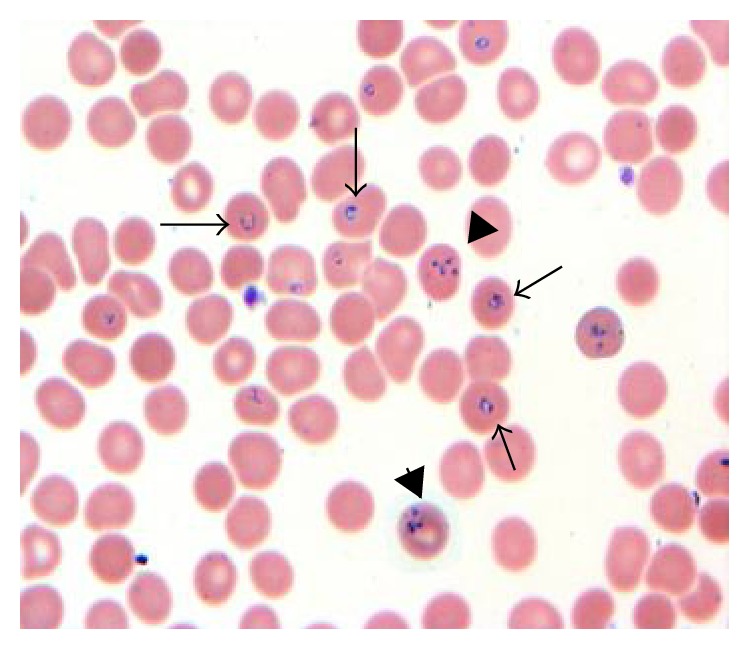
Red blood cells contain ring forms of* B. microti* (arrow) and tetrad (Maltese cross, arrow head), 1000x.

**Figure 2:**
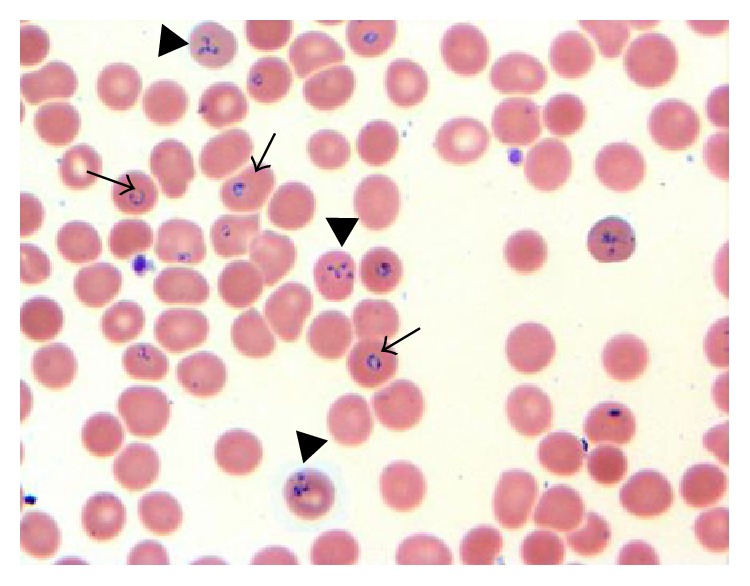
Red blood cells contain ring forms of* B. microti* (arrow) and tetrad (Maltese cross, arrow head), 1000x.

A peripheral smear was done that showed red blood cells contain ring and tetrad forms consistent with* B. microti* with 9.8% parasitemia (Figures 1 and 2). PCR for* B. microti *was positive in serum giving us definite species identification. He was started on azithromycin 500 mg/d orally on day one and then 250 mg/d from next day and atovaqoune 750 mg orally every 12 hours. Over the next day, he continued spiking temperature. His hemoglobin decreased further from 13.2 gm/dL on admission to 9.3 gm/dL on day four and he continued being symptomatic with high temperatures, chills, and sweating. A repeated parasite count revealed 16% parasitemia. Due to ongoing hemolysis and increasing parasite count, he was upgraded to the intensive care unit for red blood cell exchange therapy while his antibiotics were changed to IV clindamycin 600 mg orally every 8 hours and oral quinine 650 mg orally every 8 hours. After one session of exchange therapy, he reported feeling better. His hemoglobin stabilized around 9.3 over the following two days. His LDH decreased to 436 IU/L, his bilirubin normalized, and parasites were not detected at the end of fifth day. He was discharged on 8th day of admission on total of 10 days of oral antibiotic with close outpatient infectious disease follow-up.

## 3. Discussion

Human babesiosis is a disease caused by protozoa that invade mammalian red blood cells and reproduce within them. In 2013, the CDC was notified of 1.762 cases of babesiosis among the 27 states in USA. Severity of babesiosis depends on its host's characteristic. Age and immunocompetence are probably among the main determining factors. The most severe infections occur mainly in the elderly, splenectomized, or immunocompromised hosts.

Cases of severe* B. microti* are rare in patient with intact spleen and hence it was not clear why this patient had such an aggressive clinical course. Spleen destroys RBCs containing the parasites from blood. Therefore, asplenic individuals have increased risk for overwhelming infection and sepsis. Both humoral immunity and cellular immunity are required to fight against babesiosis. It is proposed that humoral immunity prevents infection by binding and neutralizing parasites before they invade target cells. Innate immune system is involved in controlling the growth rate and the extent of parasitemia while resolution requires T lymphocytes, specifically the CD4^+^IFN-*γ* producers which degrade intraerythrocytic parasite. The role of immunity against* B. microti* was studied in mice which showed necessity for CD4^+^ T cells and IFN-*γ* in conferring immunity against infection [1]. Unlike immunocompetent mice, SCID mice (severe combined immunodeficient mice), which lack the lymphocytes necessary for adaptive immunity, developed high degree of parasitemia which was markedly reduced by transfer of naive splenocytes. This study explains the importance of spleen in clearing the parasite.

Few cases have been reported in the literature where patient with intact spleen and no evidence of immunosuppression developed severe babesiosis. Three reported cases (2 in France and one in Spain) presented with severe flu like symptoms in immunocompetent hosts and respond to oral antibiotic. In all cases,* Babesia divergens* was identified [2, 3]. Some rare cases are seen in the United States, mostly confined to healthy individuals aged ≥50 years. One case was reported on a 59-year-old immunocompetent male presenting with ARDS-like picture in US. The patient was treated with atovaquone and azithromycin for seven days and was extubated successfully [4]. Dorman et al. reported a case 58-year-old previously healthy man, infected by* B. microti*, presenting with severe disease characterized by severe anemia, DIC, and renal and respiratory failure. No clinical improvement was seen despite him being on dual antibiotic therapy with clindamycin and quinine. Whole-blood exchange transfusion was performed on second day to lower the parasite load and replace the patient's plasma. He showed marked resolution of parasitemia and slow, general improvement [5]. Dacey et al. reported the first case of overwhelming septic shock without respiratory involvement due to babesiosis in a healthy patient with an intact spleen [6]. As in our case, why these patients developed such fulminant course is not clear and hence other host factors that affect the course of* Babesia* need further study. Many studies have shown age as clear risk factor for severe* B. microti* infection in humans. 139 patients hospitalized with babesiosis in New York State were found to have mean age of 62.5 at first hospitalization [7]. To determine the relation between age and severity, a study was done in mice using clinical isolate of* B. microti*.* The study concluded that there is* age associated decline in immune protection conferred by spleen cells. This may contribute to the loss of resistance to the parasite as we age. Also the study found that there was clear difference in susceptibility in different genetic strains of mice of same age. And it concluded that age-associated decline in resistance to* B. microti* is genetically determined [8]. Thus, further study was required to understand relation between genes and resistance or susceptibility to* B. microti*. This was also shown in a study done in inbred mouse strains that showed genetic influence on the severity of infection. Certain strain of mice as C57BL/6 were found to be highly resistant to babesiosis, in contrast to the susceptibility demonstrated by A/J, C3H, 129, and BALB/c strains [9]. This possibility of genetic susceptibility is yet to be studied in humans as this may explain why some people with intact immune system develop severe infection while others get away with mild forms. A detailed immunological study is however not required neither recommended in any immunocompetent patients presenting with severe parasitic infection including* Babesia*.

Other factors that may determine the severity of* Babesia* infection were also studied in various studies. In a chart review of patients admitted with babesiosis in a hospital in long island over the period of 13 years, it was found that about 94% of patients had some comorbid conditions; among them about 34% had splenectomy. Severe anemia defined as a hemoglobin level less than 10 g/dL was the only factor associated with the presence of complicated babesiosis. 20.6% underwent exchange transfusion and only high parasitemia of more than 10% was significantly associated with the likelihood of undergoing the transfusion [10]. This study corelates with our case as both hemoglobin less than 10% and high parasitemia of more than 10% were seen in this patient. 186 cases of* Babesia* were reported in New York State over a decade in the years 1982 to 1993. Among them 77.4% were hospitalized. Demographic characteristics of the hospitalized patients showed that majority of them were old male patients with history of chronic disease [7].

As referenced above, our case of severe babesiosis in an immunocompetent host could be secondary to number of other underlying factors. Age related loss on immune system and possible genetic susceptibility probably play role in determining the severity of infection.
